# Multiscale Microparticle Trapping Through Tapered-Fiber Structures and Refractive-Index Modulation

**DOI:** 10.3390/bios16100557

**Published:** 2026-10-03

**Authors:** Xiaoyue Chen, Yubin Xiong, Hanbing Zhu, Yinhong Su, Leyi Zheng, Mu Ku Chen, Meng Zhang, Lip Ket Chin, Wu Zhang

**Affiliations:** 1 School of Physics and Material Science, Guangzhou University, Guangzhou 510006, China; xiaoyue.chen@e.gzhu.edu.cn (X.C.); ly.zheng@e.gzhu.edu.cn (L.Z.); 2 Department of Electrical Engineering, City University of Hong Kong, Hong Kong SAR 999077, China; yubixiong2-c@my.cityu.edu.hk (Y.X.); mkchen@cityu.edu.hk (M.K.C.); 3 School of Electronics and Communication Engineering, Guangzhou University, Guangzhou 510006, China; 32519500060@e.gzhu.edu.cn (H.Z.); zhangmeng@gzhu.edu.cn (M.Z.); 4 School of Economics and Statistics, Guangzhou University, Guangzhou 510006, China; 32419700006@e.gzhu.edu.cn; 5 Department of Electrical and Electronic Engineering, The Hong Kong Polytechnic University, Hong Kong SAR 999077, China; 6 Research Institute for Quantum Technology, The Hong Kong Polytechnic University, Hong Kong SAR 999077, China

**Keywords:** fiber optical tweezers, tapered optical fiber, particle trapping, refractive-index modulation, trapping distance

## Abstract

Tapered optical fibers reshape optical fields in confined spaces, enabling versatile microparticle trapping. This study establishes a continuous framework spanning near-field confinement, far-field extension, and environmental modulation by comparing flame-tapered and machine-pulled fibers. Flame tapering produces sharp tips with micrometer-scale foci; taper angles of 24°, 40°, and 60° yield focal positions of 0.2, 5.1, and 7.0 µm, respectively. A 980 nm laser is used, and stable trapping and probe-guided transport of 20 µm polystyrene microspheres were achieved at a threshold of 84 mW, with dual-particle trapping demonstrated via microlens-induced secondary potential wells. In contrast, machine-assisted tapering generates smooth end faces and long transition regions, extending working distances to 67, 122, and 163 µm. A long-transition probe further produced dual axial potential wells that stably confined 5 µm microspheres at 143 and 286 µm at powers above 500 mW. Environmental modulation revealed that increasing the external refractive index from 1.33 to 1.36 shifted the simulated focal positions from 40 to 55 µm, while ethanol–water evaporation experimentally reduced the trapping distances from 353 to 236 µm. These results show that fiber geometry defines the trapping range, whereas refractive index tunes the precise trapping position, offering a compact platform for multiscale manipulation with potential applications in cell positioning and sorting in liquid environments.

## 1. Introduction

Optical tweezers exploit the gradient force generated by a nonuniform optical field to confine microscopic particles, providing a versatile platform for noncontact trapping, positioning, and transport. Since Ashkin and co-workers first demonstrated radiation-pressure-driven motion and single-beam gradient-force trapping [1,2], optical manipulation has become a robust technique for probing microparticles. In dielectric systems, the optical-field gradient restores particles toward high-intensity regions, while scattering forces drive them along the beam axis. Stable trapping requires a recoverable spatial balance between these contributions [3,4,5]. Over the past decades, optical tweezers have evolved into powerful tools for the precision manipulation and mechanical measurements of living cells, DNA–protein complexes, colloids, and micro- and nanostructures [4,6,7,8,9,10,11]. In biological contexts, their noncontact, minimally invasive operation in liquids has enabled single-cell trapping, separation, controlled rotation, and mechanical-response measurements [6,9,12,13,14,15,16].

Conventional optical tweezers rely on high-numerical-aperture objectives, but their bulk and limited working distance restrict their use in droplets, microchannels, and other confined environments. Fiber-based tweezers address these limitations by integrating light delivery, focusing, and manipulation into a single probe. Such systems enable direct insertion into samples, particle transport via fiber motion, and seamless integration with microfluidics [17]. Recent advances include far-field fiber-tip tweezers that achieve stable nanoparticle trapping [18,19,20], dual-fiber systems with 3D-printed Fresnel lenses for efficient three-dimensional confinement [21,22,23], and tapered or multimode probes for enhanced transport, multiwell manipulation, and biological trapping [10,11,12,13,24,25,26,27,28,29,30].

With progress in fusion tapering and end-face microstructuring, fiber probes now support multiple potential wells, directional transport, and living-cell manipulation [10,15,16]. For biological applications, an ideal probe must generate a high-gradient optical field near the tip for precise local positioning, while maintaining stable confinement farther away to minimize mechanical disturbance and interfacial effects [9,11,15,16]. Fresnel structures fabricated on fiber end faces can form focal regions 5–10 µm from the tip [11]. In contrast, graded-index structures and end-face microcavities extend the range of manipulation to hundreds of micrometers [31]. However, most studies validate individual structures in isolation, with limited direct comparison of geometrical morphology, output fields, and trapping distances across tapering methods. Furthermore, once fabricated, a probe’s optical-field distribution and working distance are essentially fixed, leaving optical power as the only adjustable parameter.

To address these limitations, this work introduces a multiscale manipulation strategy that combines structural fiber tapering with environmental refractive-index modulation. Alcohol-lamp tapering produces micrometer-scale near-field traps, whereas machine tapering with lensed end faces achieves hundred-micrometer-scale far-field confinement. Crucially, by modulating the surrounding medium’s refractive index, the stable trapping position can be dynamically adjusted, transforming an environmental factor from a passive condition into an active dimension for distance regulation. In this framework, the fiber geometry dictates the accessible trapping range, while the medium’s refractive index tunes the precise equilibrium position. We demonstrate this mechanism through comparative studies of short alcohol-lamp tapers and long machine-fabricated tapers, examining near-tip, far-field, and dual-well trapping in water, ethanol, and evaporating binary mixtures.

## 2. Short-Range Trapping with an Alcohol-Lamp-Tapered Fiber

### 2.1. Simulation of the Output Field of a Flame-Tapered Optical Fiber

To clarify the relationship between probe geometry and the near-field focal position, a two-dimensional model of a tapered fiber immersed in a liquid was constructed using the finite-difference time-domain (FDTD) method implemented in Ansys Lumerical. The model included the fiber core, cladding, external medium, and open boundaries. Standard material parameters for a single-mode silica fiber were assigned to the core and cladding, while the external domain matched the refractive index of the experimental liquid. A 980 nm port source launched the fundamental mode along the optical axis. Scattering boundary conditions suppressed artificial reflections at the domain edges.

The mesh was locally refined in the taper, end face, and expected focal region to capture fine-scale field variations. In contrast, a coarser mesh was used in uniform regions farther away. After assigning materials, source, and boundaries, the steady-state electric field was solved, and normalized axial intensity profiles were extracted to compare focal positions and peak intensities across taper geometries (Table 1).

**Figure 1:**
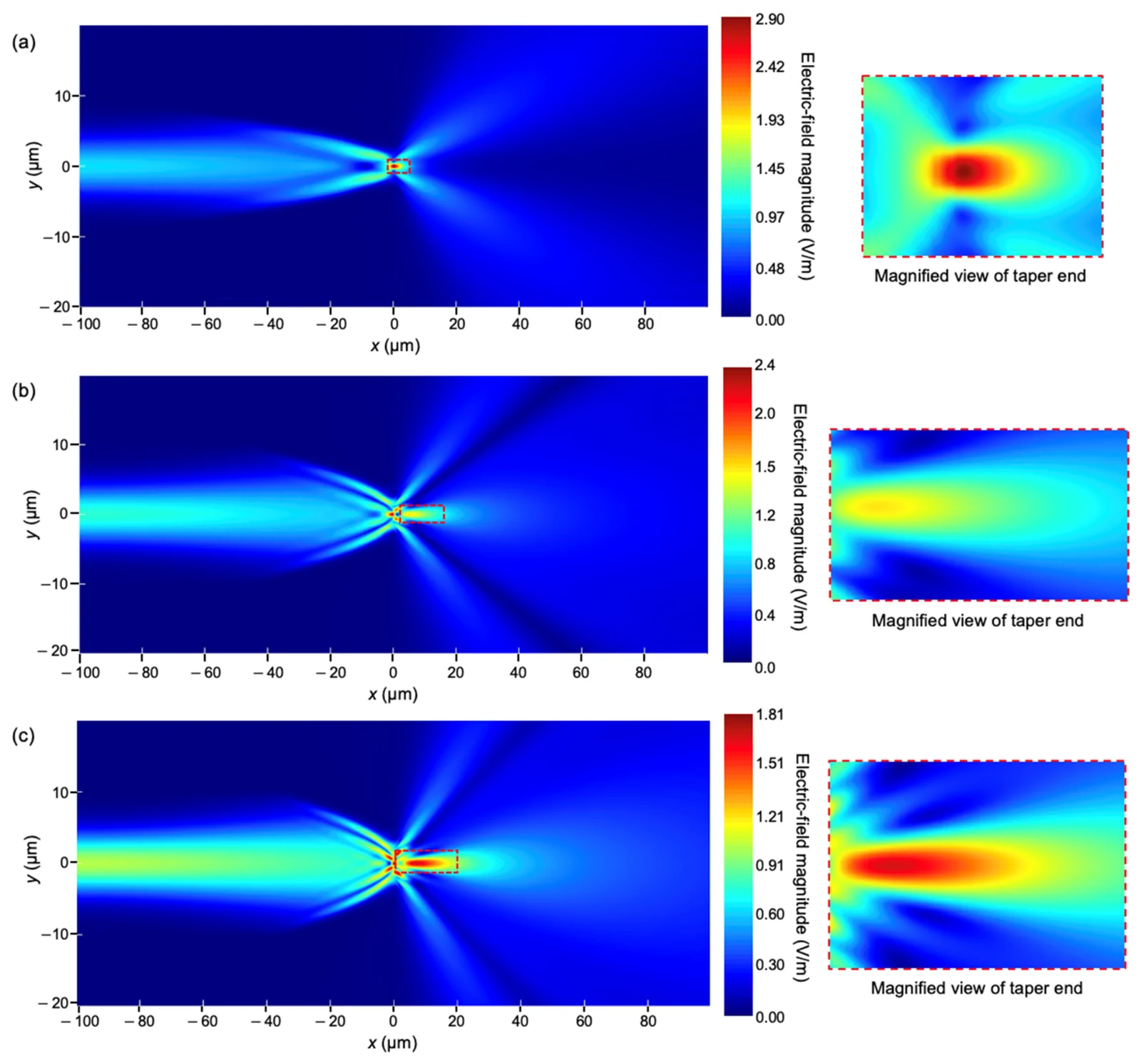
Simulated electrical fields of probes with different taper angles: (**a**) 24°, (**b**) 40°, and (**c**) 60° for Probes A, B, and C, respectively.

**Figure 2:**
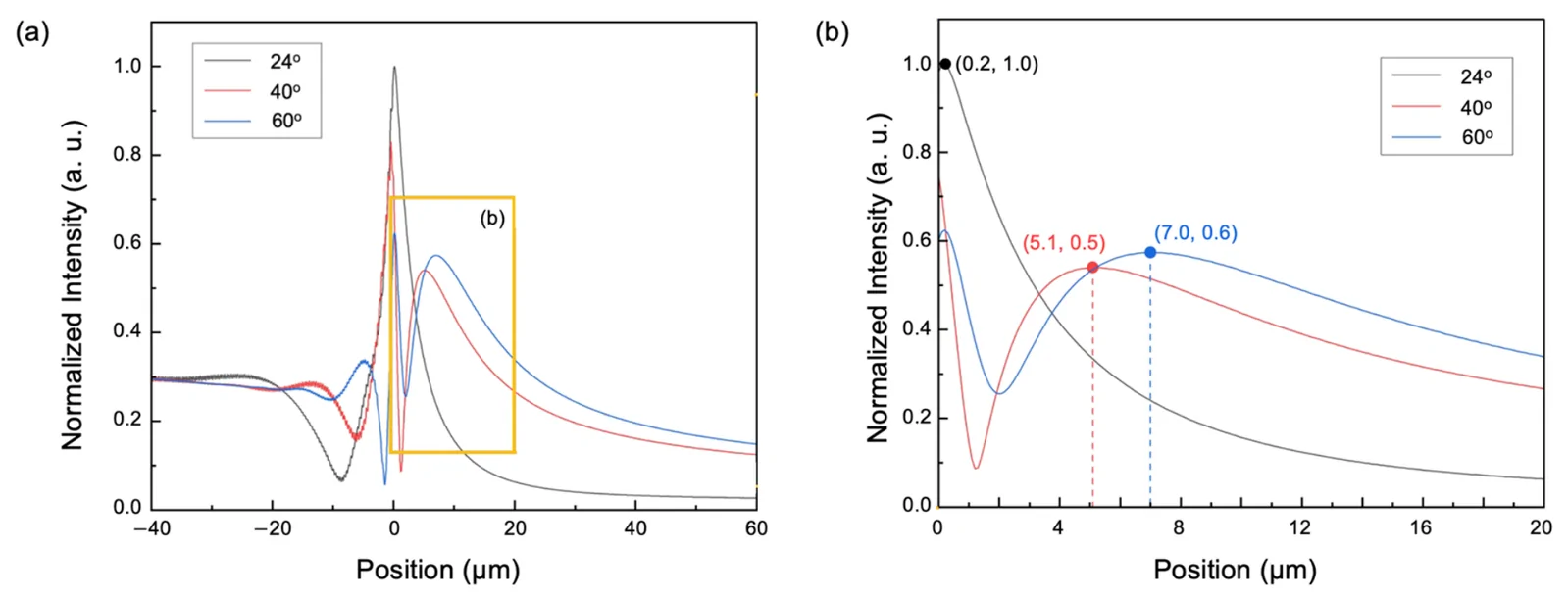
Axial normalized intensity profiles of probes with different taper angles (**a**) for the range from −40 µm to 60 µm, and (**b**) 0 to 20 µm.

Three models (Probes A–C) were simulated using these parameters (Figure 1). All probes guide energy through the taper and produce localized convergence at the end face, but the taper angle strongly influences propagation and leakage. For Probe A, after emerging from the tip, the beam forms a convergent spot approximately 0.2 µm from the end face (Figure 2), after which the field rapidly diverges, producing an elongated axial intensity distribution. For Probe B, partial energy leaks through the sidewall as lateral radiation. Its convergence is therefore weaker than that of Probe A, and the focus is located approximately 5.1 µm from the tip. For Probe C, which has the largest taper angle, the focal spot is approximately 7.0 µm from the tip.

As the taper angle decreases, the optical field becomes more strongly localized near the tip, yielding higher peak intensity but shorter working distance. Increasing the taper angle shifts the focus farther from the end face, extending the interaction range but reducing peak intensity. Thus, probe geometry must balance near-field confinement against usable working distance. Overall, the output field of a flame-tapered fiber is concentrated within several micrometers of the end face, making this structure particularly suitable for precise near-tip manipulation of microparticles.

**Figure 3:**
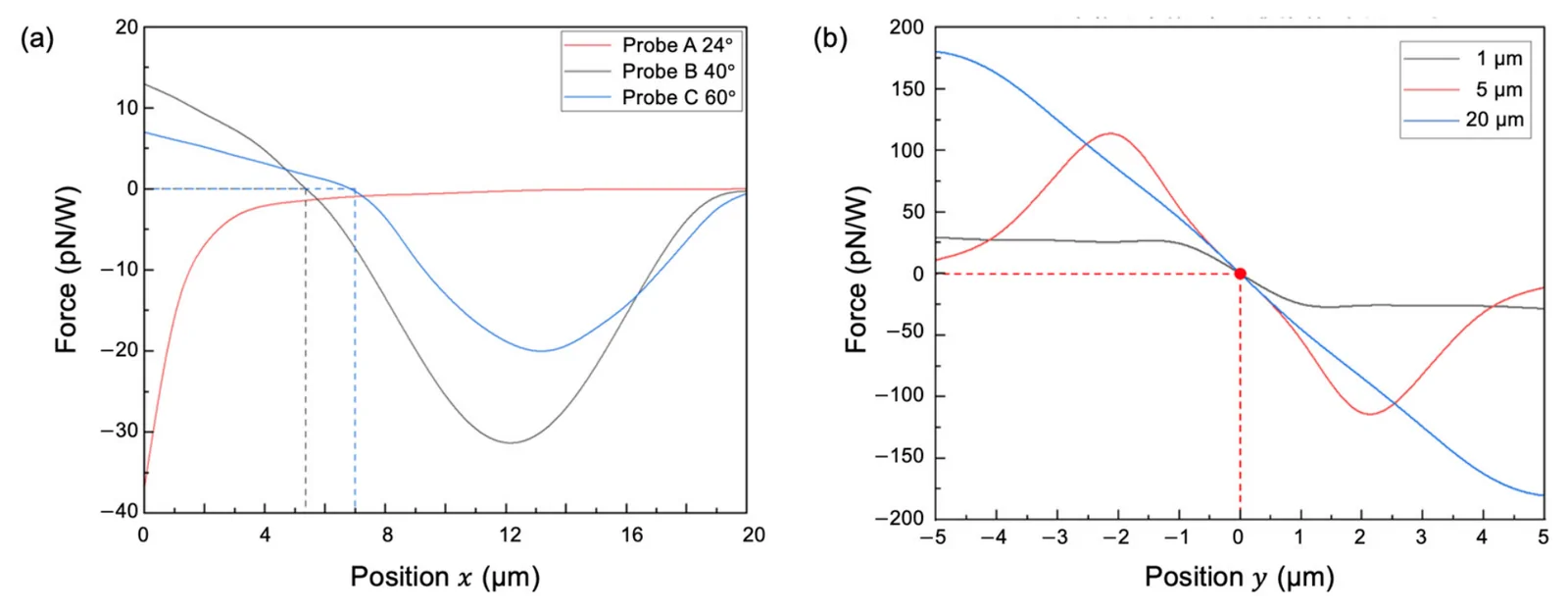
Calculated optical force per unit input power exerted by tapered fibers on polystyrene (PS) microspheres. (**a**) Longitudinal force acting on a 1 µm PS microsphere. (**b**) Transverse force produced by Probe B (θ = 40°) on PS spheres with diameters of 1, 5, and 20 µm.

We subsequently calculated the optical force exerted on a 1-µm-diameter polystyrene (PS) microsphere. The optical force exerted by a tapered fiber on a dielectric microsphere depends critically on the taper’s cone angle, which governs both the transverse confinement of the guided mode and the axial gradient of the evanescent field. Figure 3 compares the calculated optical force per unit input power across three tapered probes with cone angles of 24° (Probe A), 40° (Probe B), and 60° (Probe C). Along the fiber axis (Figure 3a), the 1 µm microsphere experiences distinct force profiles depending on *θ*. The sharpest probe (24°) generates the strongest peak force at the tip (approximately −35 pN/W at *x* = 0); however, this attractive (negative) force decays steeply with distance and remains negative over the entire examined range, pushing the zero-force equilibrium position beyond *x* ≈ 7 µm. In contrast, the 40° and 60° probes exert repulsive (positive) forces of roughly 12.5 and 7 pN/W near the taper, which reverse sign at *x* ≈ 5.5 µm and *x* ≈ 7 µm, respectively. Beyond these zero crossings, both profiles exhibit a pronounced attractive well, reaching minima of approximately −32 pN/W at *x* ≈ 12.5 µm (*θ* = 40°) and −20 pN/W at x ≈ 15 µm (*θ* = 60°). Consequently, the 40° probe yields the highest longitudinal trapping force and the deepest potential well, demonstrating an optimal cone angle governed by the trade-off between beam divergence and the axial intensity gradient.

Perpendicular to the fiber axis (Figure 3b), the transverse force produced by the 40° probe exhibits a strong dependence on particle size. For a 1 µm microsphere, the transverse force is relatively weak and nearly constant, approximately 25 pN/W for *y* < 0 µm and −25 pN/W for *y* > 1 µm, with a sign reversal near the fiber axis. For the 5 µm microsphere, the force profile becomes bipolar, featuring a positive peak of 120 pN/W at *y* ≈ −2 µm and a negative peak of −120 pN/W at *y* ≈ 2 µm. For the 20 µm microsphere, the peak force reaches ±180 pN/W at *y* = ±5 µm and varies almost linearly across the fiber axis, crossing zero at *y* = 0. The transverse optical force thus increases superlinearly with particle diameter.

### 2.2. Fabrication of Flame-Tapered Optical Fibers

**Figure 4:**
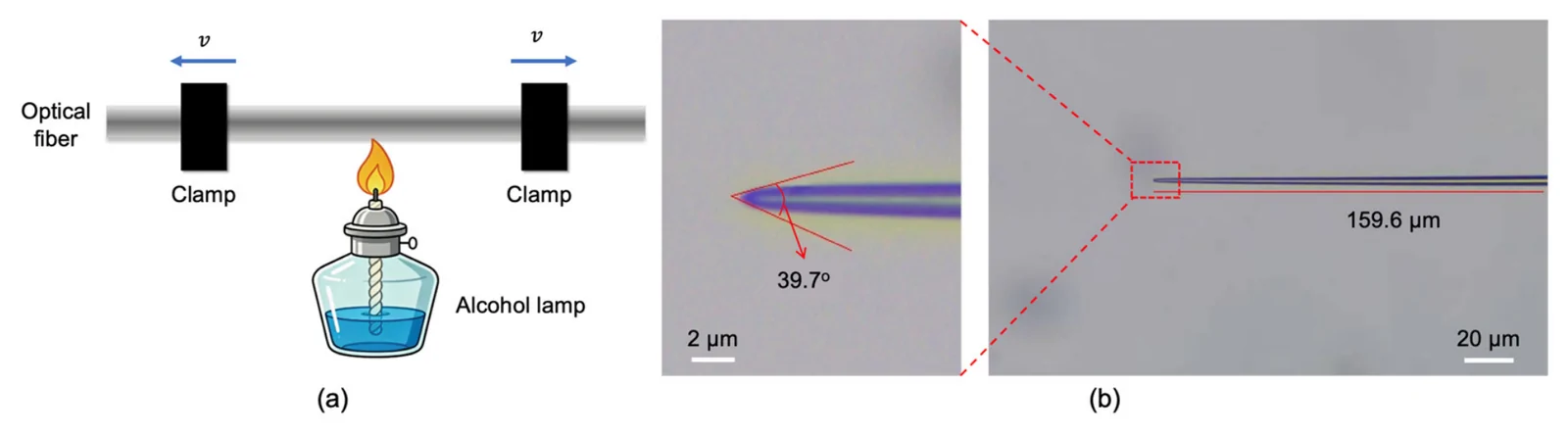
Fabrication and structure of the alcohol-lamp-tapered fiber. (**a**) Local flame heating and bidirectional pulling of the bare fiber. (**b**) Micrograph of the sharp tapered probe.

Alcohol-lamp flame tapering combines localized heating with controlled bidirectional pulling to form single-ended fiber probes. After removing the coating from a single-mode fiber and cleaning the exposed surface, the bare fiber was mounted between two clamps (Figure 4a). The fiber was preheated for 5–10 s to soften it, after which the clamps were moved in opposite directions at a constant speed. Pulling speed determined the rate of diameter reduction, while the heated-zone length and flame position jointly defined the taper length and angle. Once a narrow waist was formed, the fiber was cleaved at the waist to yield a single-ended tapered probe, followed by an electrical discharge treatment to smooth the fiber end. Although simple and flexible, this method is sensitive to local temperature distribution and the consistency of manual pulling, which directly affect taper morphology.

The microscopic image in Figure 4b shows that the fabricated probe exhibits a continuous transition region, a taper angle of approximately 39.7°, a tip diameter of approximately 9 μm, and a taper length of approximately 159.6 μm, with no visible fractures or large-scale surface defects. Before use, the probe tip was immersed in anhydrous ethanol and ultrasonically cleaned for 30 s, then rinsed with deionized water. The cleaned probe was used promptly to minimize dust deposition and residual contaminants that could perturb the end-face optical field.

### 2.3. Single-Microsphere Trapping and Manipulation

The experimental system employed a semiconductor laser (center wavelength: 980 nm) and 20 µm PS microspheres as model particles. Laser light was coupled into a single-mode fiber and delivered to the flame-tapered probe, which was mounted on a micropositioning stage for precise alignment with target microspheres under microscopic observation. Before each experiment, the stock suspension was diluted to 0.05 mg/mL in the droplet medium and ultrasonicated for 5 min to minimize aggregation. A 100–200 µL aliquot was deposited onto a hydrophobic polydimethylsiloxane-coated glass substrate, forming a spherical-cap droplet (diameter ~5–10 mm). After resting for 30 s to stabilize the droplet shape and internal flow, the probe was carefully inserted into the liquid, and the input power was increased stepwise. This sequence minimized interference from probe insertion, free-surface vibration, and particle clustering.

**Figure 5:**
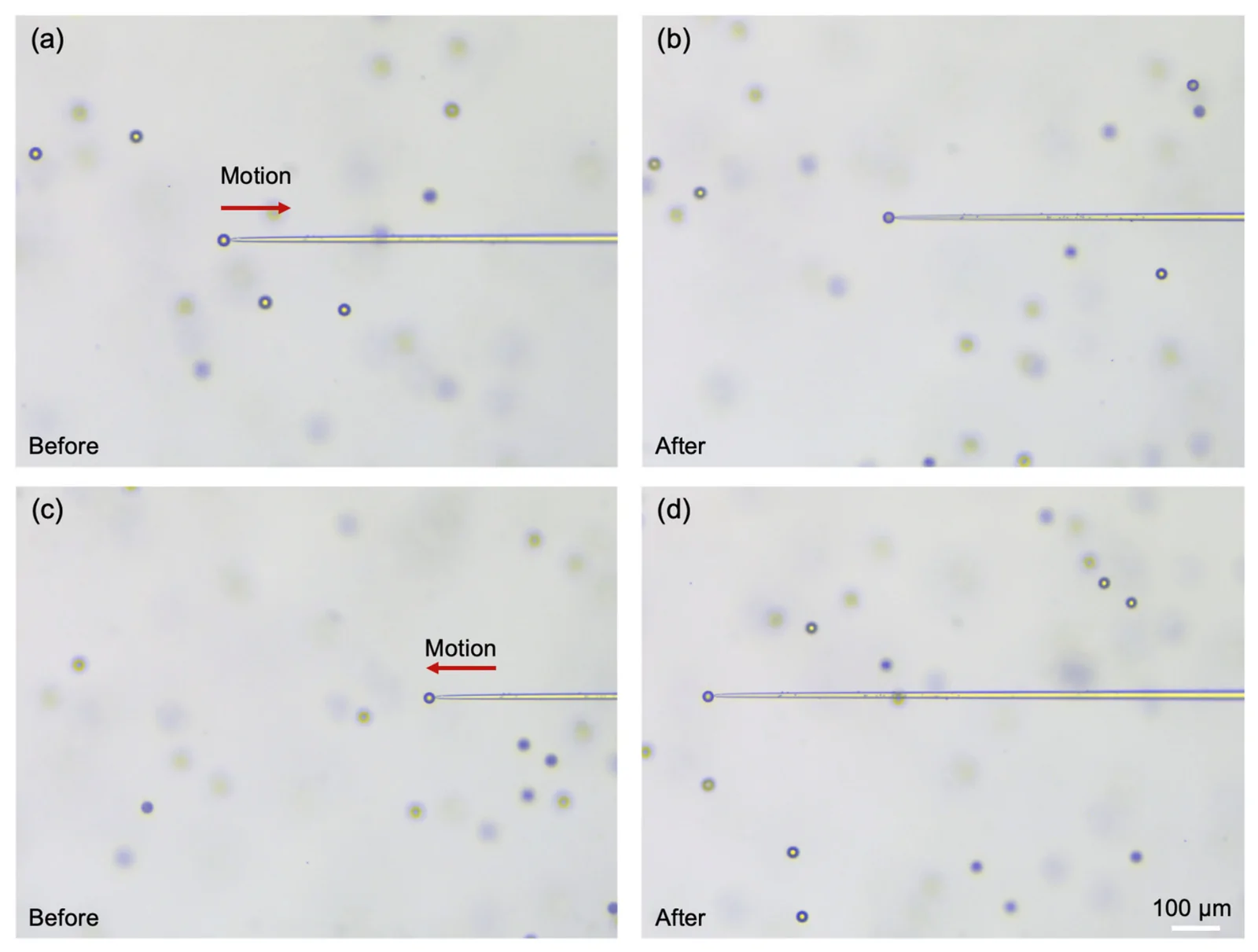
Lateral transport of a trapped microsphere with the probe: (**a**,**b**) motion of the probe to the right; and (**c**,**d**) motion to the left after reversal. The microsphere remains confined within the near field of the tip.

To evaluate confinement stability during probe motion, the fiber was translated transversely after trapping was established. As shown in Figure 5, the microsphere remained within several micrometers of the fiber tip, consistently following probe motion to the left and right without transverse escape (see Supplementary Movie S1). The nearly constant particle–tip separation demonstrates that the trap provides sufficient lateral confinement to resist hydrodynamic disturbances during translation.

**Figure 6:**
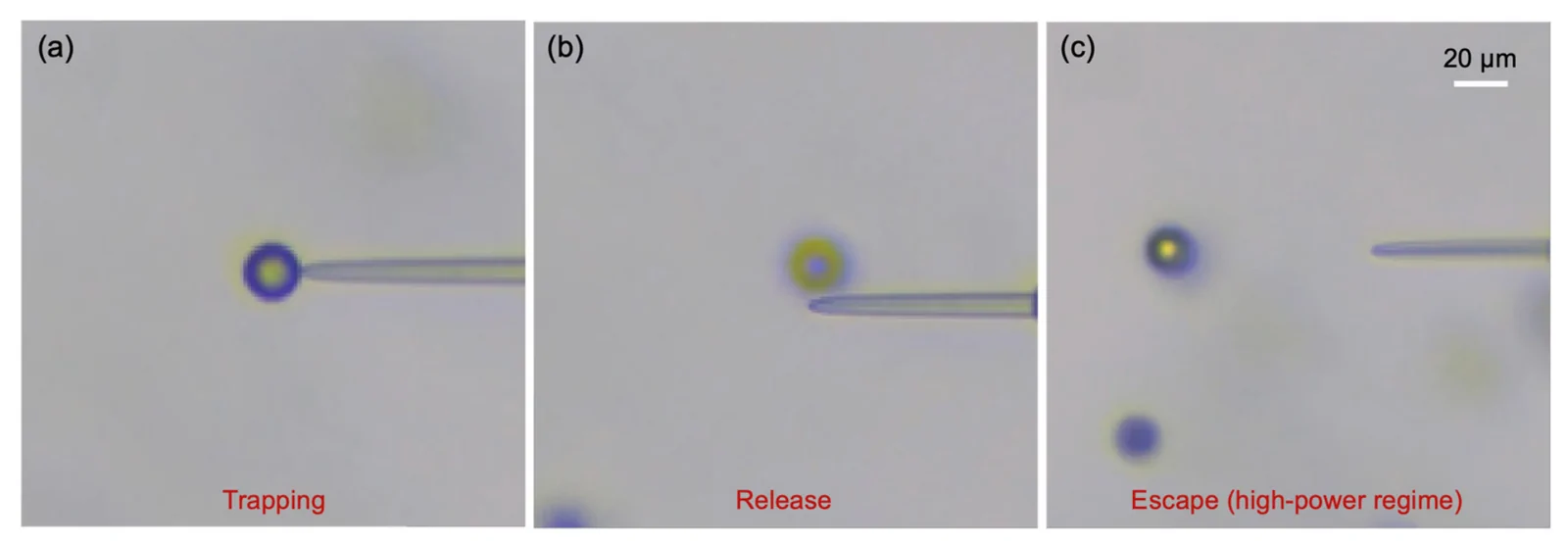
Laser on or off and power responses of a 20-µm PS microsphere at 980 nm: (**a**) stable trapping at 84 mW; (**b**) release after the laser is switched off; and (**c**) escape in the high-power critical regime.

Figure 6 illustrates the on/off response and power dependence of single-microsphere trapping. With the laser off, the microsphere exhibited Brownian motion and slow drift within the droplet. Its relatively large diameter reduced fluctuation amplitude, but its position remained random. When the laser was switched on, and the power increased to 84 mW, the microsphere was drawn toward the fiber tip and confined within ~1–2 s. Switching off the laser released the particle, which escaped laterally. At the critical power of 122 mW, particle oscillation increased abruptly, and further increases in power caused escape, typically after a short axial displacement followed by lateral release (see Supplementary Movie S2).

Table 2 summarizes that 84 mW is the stable-trapping threshold in this experiment, and approximately 84–120 mW constitutes a relatively stable operating range. Below this range, the gradient force is insufficient to overcome particle disturbances continuously. Above 122 mW, particle oscillation and the escape probability increase rapidly. Thus, higher input power does not necessarily improve stability; practical operation requires balancing enhanced optical forces against photothermal effects. The onset of oscillatory motion at the critical power (~122 mW) is attributed to the interplay between optical and thermal forces. As the input power increases, enhanced scattering forces and laser-induced heating of the surrounding liquid generate thermocapillary flow and convection, which perturb the equilibrium established by the gradient force. These competing effects destabilize confinement, leading first to oscillations and ultimately to particle escape. It should be noted that the quoted input powers refer to the laser output before fiber coupling. Direct measurements at the tapered fiber tip indicate that only ~20–30% of the launched power is transmitted to the probe apex, depending on taper geometry. Therefore, the effective optical power available for trapping is substantially lower than the nominal input.

This power-dependent optical trapping can be compared with strategies that demonstrate polarization-controlled switching among optical-trapping, propulsion, and stability regimes [32,33,34,35]. In those schemes, modulating the polarization state switches between stable trapping and forward propulsion at constant optical power. In contrast, the transition observed in our approach is primarily governed by the competition between increasing axial scattering forces and photothermal disturbances. While polarization modulation offers rapid, athermal state switching, our power-regulated approach provides a straightforward, single-parameter operational window that circumvents the need for specialized polarization-maintaining components or complex wavefront modulators.

Although stable optical manipulation was experimentally verified, quantitative calibration of the trap stiffness via Brownian motion tracking was limited by the temporal resolution of our imaging system. Future work will focus on integrating high-bandwidth tracking to measure trap stiffness and benchmark it directly against our simulation results.

### 2.4. Optical-Field Perturbation Induced by a Microsphere

**Figure 7:**
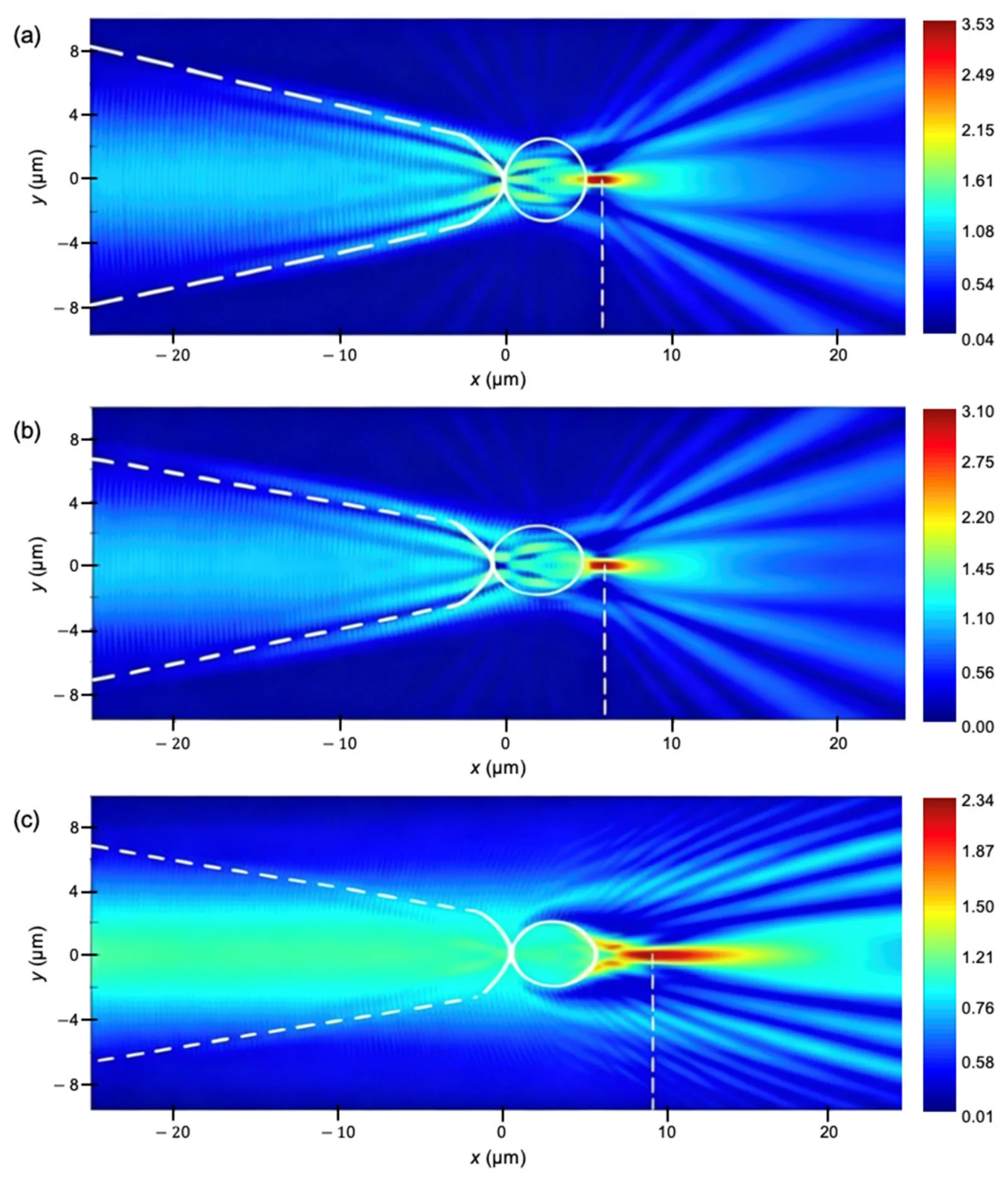
Perturbation of the tapered-fiber output field by a microsphere in (**a**) water, (**b**) ethanol, and (**c**) glycerol, respectively. Refraction, scattering, and the microlens effect reshape the optical field behind the particle.

A trapped microsphere not only responds to the incident optical field but also reshapes the downstream optical field, acting effectively as a secondary optical element. To analyze this process, a PS microsphere (n=1.59) was introduced in the output field of the flame-tapered probe, and the resulting field distributions were compared in water (n=1.33), ethanol (n=1.36), and glycerol (n=1.47) (Figure 7). At the particle interfaces, incident light is refracted and partially scattered; interference between transmitted and scattered components produces a modified intensity distribution downstream of the sphere. Because the PS microsphere has a higher refractive index than the surrounding liquid, it behaves as a microlens that reconverges transmitted light and generates a high-intensity region behind the particle.

The position, peak intensity, and sidelobe structure of this secondary focus depend strongly on the relative refractive-index contrast between the PS microsphere and the medium. In glycerol, where the refractive-index contrast is smallest (∆n=0.12), the downstream high-intensity region extends farther from the particle. It spans roughly 5 µm in width, indicating that the external refractive index changes both the secondary convergence position and the focal-region dimensions. A larger relative-index contrast produces stronger microlens focusing (for water and ethanol), whereas a smaller contrast (glycerol) broadens and weakens the downstream high-intensity region.

**Figure 8:**
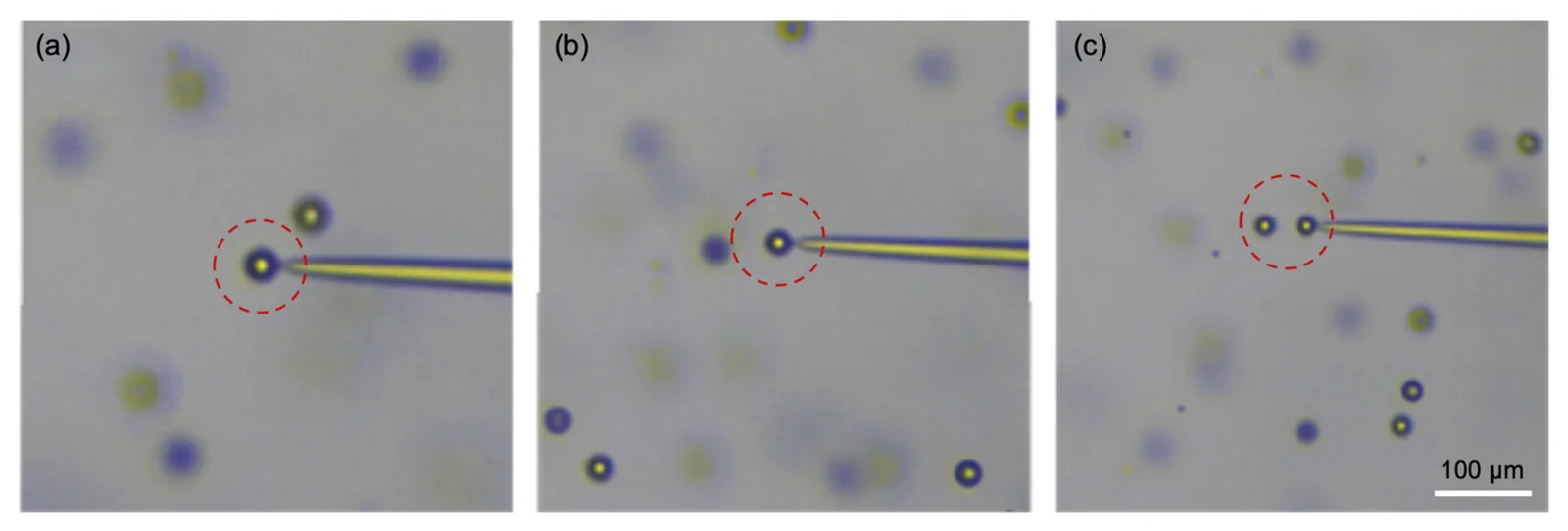
Dual-microsphere trapping: (**a**) first microsphere enters the primary potential well; (**b**) probe is moved to search for a second microsphere; (**c**) second microsphere is trapped in the secondary high-gradient region behind the first particle.

The microlens-induced redistribution of the output field can create a secondary potential well that traps an additional microsphere (see Supplementary Movie S3). Experimentally (Figure 8), after the first particle was stably trapped near the probe tip, the probe was translated to search for a second particle; when a second microsphere entered the microlens-shaped high-gradient region behind the first, it experienced a restoring gradient force and became confined in a secondary potential well. Because this secondary potential well is generated by the field perturbation of the primary particle, its depth and location are highly sensitive to particle decentering, interparticle spacing, and local fluid flow. Consequently, the secondary trap is intrinsically less robust than the primary potential well adjacent to the end face.

In practice, dual-particle operation requires careful balancing of optical forces and hydrodynamic and thermal disturbances. In our experiments, input powers of 100–120 mW provided the most favorable conditions for maintaining a stable two-particle configuration: lower powers failed to draw the second particle into the secondary focus, whereas higher powers amplified photothermal convection and scattering forces, increasing the oscillation and escape probabilities of the downstream particle. These results indicate that flame-tapered probes are particularly well suited for precise near-tip manipulation, and that achieving reliable dual-particle trapping demands a narrow operating window that balances gradient confinement against thermally induced perturbations and enhanced scattering.

## 3. Long-Range Trapping with Machine-Pulled Tapered Fibers

### 3.1. Fabrication and End-Face Treatment of Machine-Pulled Tapered Fibers

The short, heated zone of alcohol-lamp tapering concentrates softening near the flame, producing probes with short transition regions and output fields confined to a few micrometers from the end face. Reproducible fabrication of long, gradual transitions is therefore challenging with manual flame methods because flame temperature, heated length, and pulling speed are difficult to control precisely. To extend the optical interaction distance and improve structural repeatability, a programmable tapering machine was employed.

**Figure 9:**
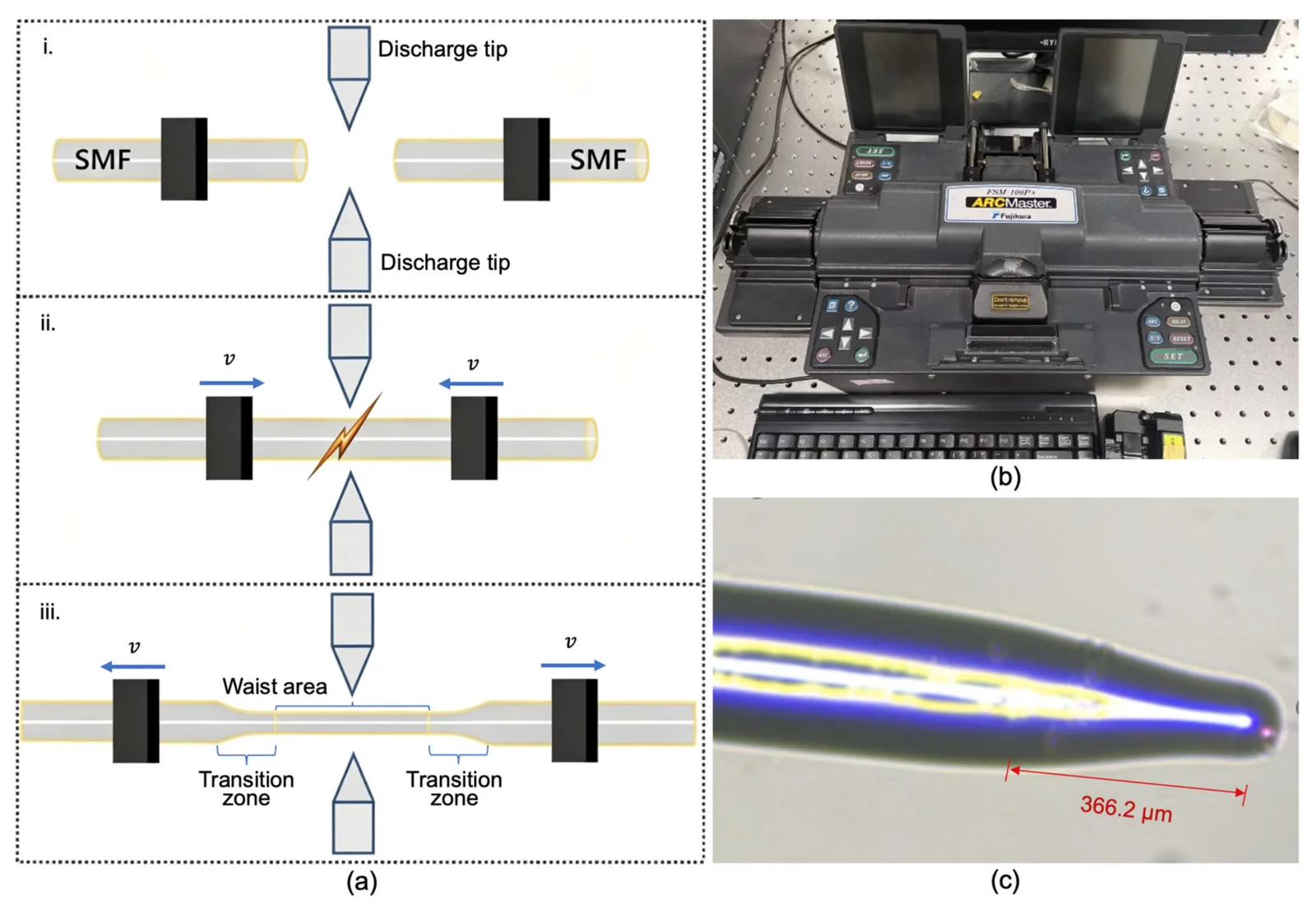
Fabrication and end-face treatment of the machine-pulled tapered fiber: (**a**) (i) fiber alignment, (ii) arc fusion, (iii) bidirectional pulling, and formation of the transition-waist-transition structure; (**b**) FSM-100P + tapering system; and (**c**) probe obtained after waist cutting and end-face arc treatment.

The fabrication procedure is illustrated in Figure 9a. First, Coatings were stripped from two single-mode fibers, and the exposed surfaces were cleaned with ethanol-wetted, lint-free cloths. The fiber ends were cleaved to produce flat faces, then aligned and positioned near the discharge region of a specialty fusion splicer (Figure 9b). Microscopic observation ensured axial alignment with the discharge electrodes. An electric arc locally softened the silica, while motors on both sides executed programmed, opposing dis-placements at preset speeds, producing a controlled transition–waist–transition geometry. Compared with flame tapering, the arc power, pulling speed, and displacement are fully programmable, enabling longer and more gradual transition regions with high reproducibility. After tapering, the fiber was severed at the waist, and one segment was retained as the probe. Because the freshly cut end can exhibit sharp edges or irregular fracture morphology, a secondary arc discharge was applied to reflow and smooth the tip, producing the lens-shaped end face shown in Figure 9c. This post-processing both reduces scattering from edge defects and extends the effective trapping distance from micrometers to the hundred-micrometer scale.

### 3.2. Optical-Field Simulation of Parabolic Tapered Fibers

**Figure 10:**
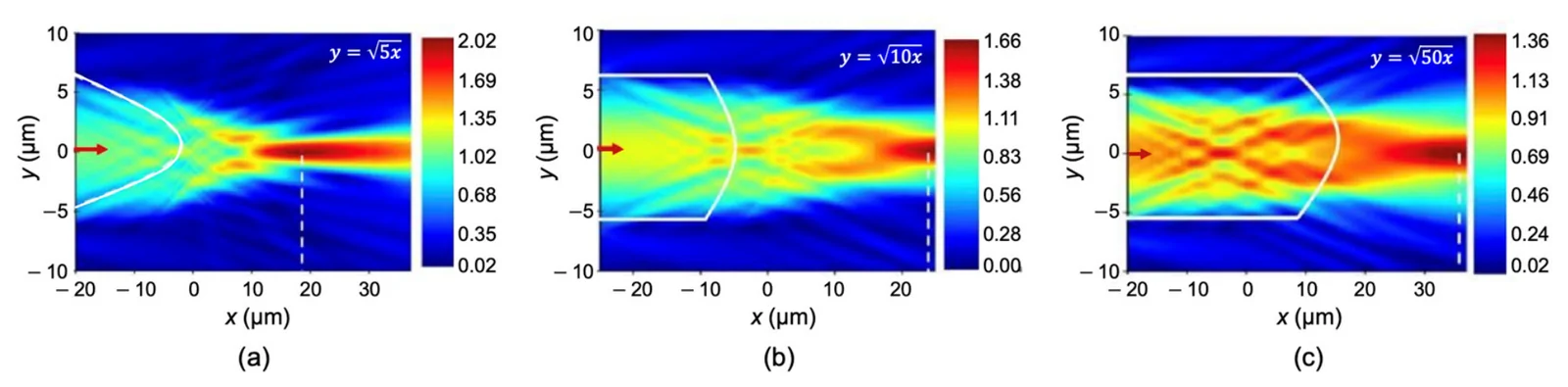
Output fields of three parabolic tapered-fiber geometries with long transition regions: (**a**) $y=\sqrt{5x}$; (**b**) $y=\sqrt{10x}$; (**c**) $y=\sqrt{50x}$. The red arrows indicate the input light direction; the solid white lines outline the fiber profiles, and the white dashed lines indicate representative focal positions.

End-face geometry strongly influences the output wavefront and focal-field distribution. To identify geometries suitable for long-range trapping, finite-difference simulations compared three parabolic end profiles described by $y=\sqrt{ax}$ with coefficients a = 5, 10, and 50 (Figure 10). The parabolic coefficient controls end curvature and thus the emerging wavefront curvature. The $y=\sqrt{5x}$ profile produces a relatively concentrated primary focus at short distances but offers limited focal-length extension. The intermediate $y=\sqrt{10x}$ configuration shifts the focus farther from the tip while retaining pronounced axial convergence, providing a favorable compromise between working distance and local intensity. Increasing the coefficient to a = 50 yields a blunter end face: the beam remains axially concentrated over a longer range, but the peak intensity decreases, and the focal region broadens. In summary, increasing the parabolic coefficient extends the effective interaction distance at the expense of local peak intensity. These simulations guided the selection of machine-pulled geometries that provide substantially longer focal distances than flame-tapered probes and are therefore suitable for long-range trapping. It should be noted that the parabolic profile is an idealized approximation used to investigate the influence of end-face curvature. The actual morphology of the mechanically tapered probe depends on fabrication parameters and was not reconstructed in detail here; therefore, the comparison between simulation and experiment is qualitative.

### 3.3. Long-Range Trapping with Tapered Optical Fibers

**Figure 11:**
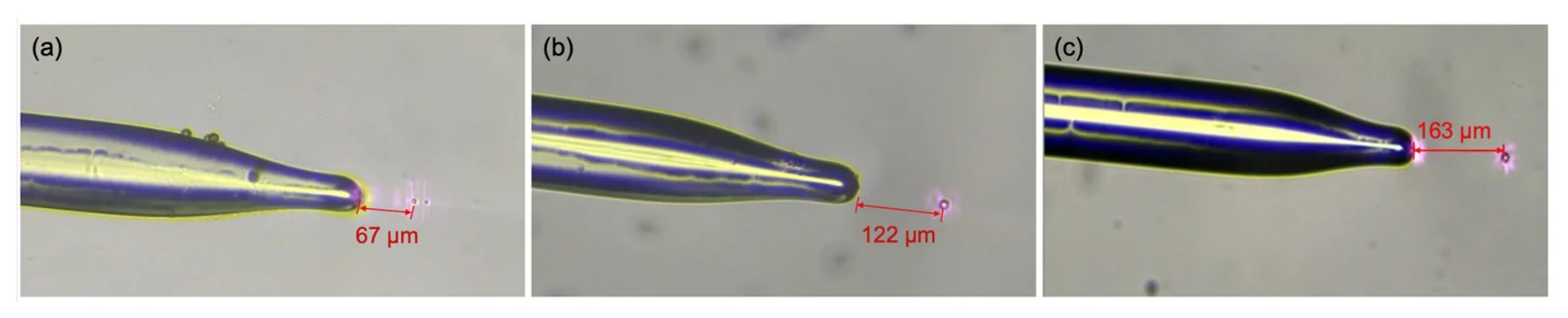
Long-range trapping with tapered fibers having different transition-region geometries: (**a**) 67 µm; (**b**) 122 µm; (**c**) 163 µm.

Microsphere-trapping experiments were performed with probes having extended transition regions and varied end-face curvatures. Using a 980 nm laser and 20 µm polystyrene microspheres, all tested machine-pulled probes exhibited stable axial confinement at distances well away from the end face. Representative trapping distances measured in Figure 11 were approximately 67 μm, 122 μm, and 163 μm, demonstrating that programmable tapering can reproducibly generate probes capable of long-range optical confinement.

### 3.4. Dual-Focus Trapping with a Long-Transition-Region Tapered Fiber

**Figure 12:**
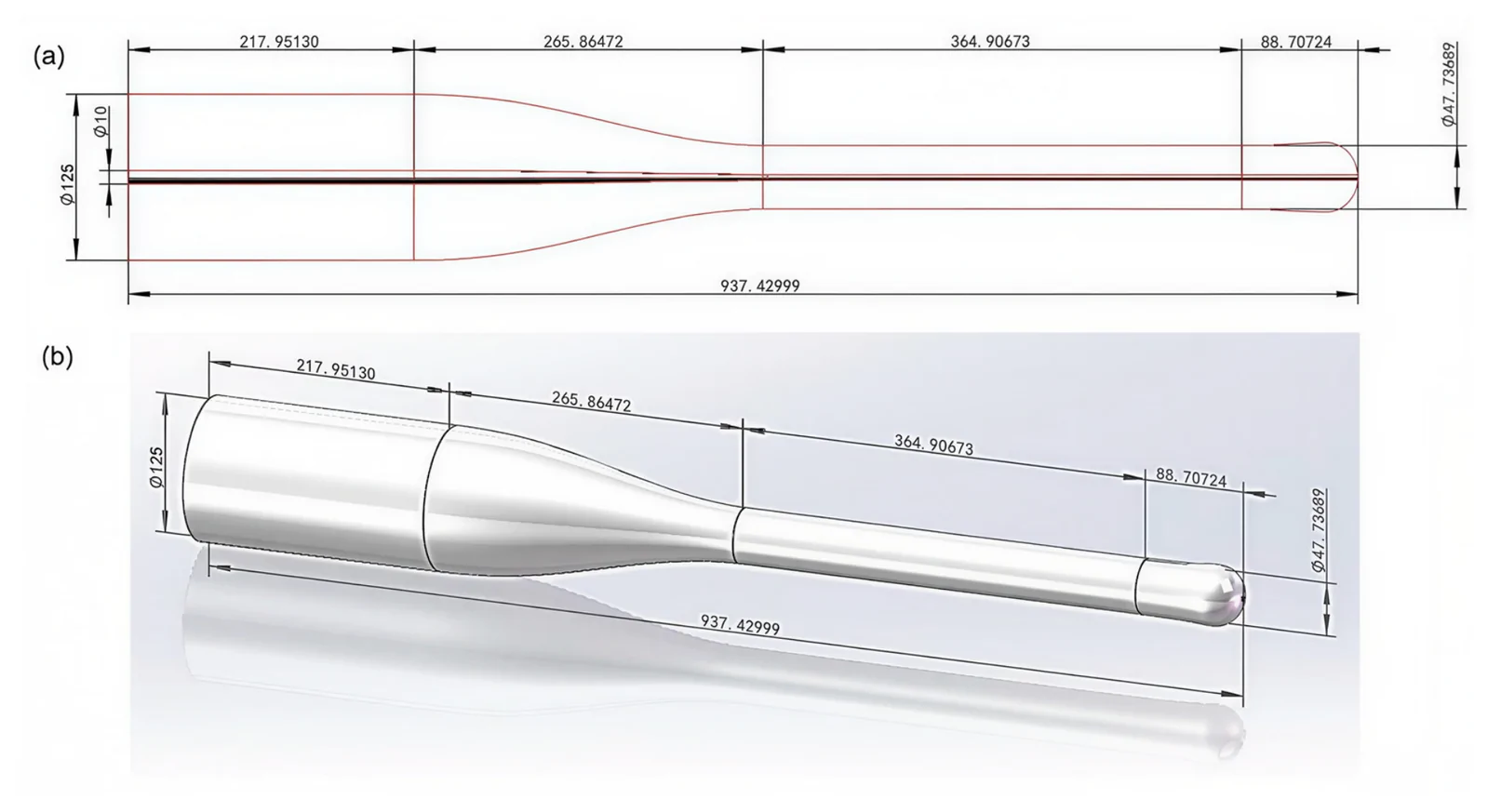
Geometrical dimensions and structural model of the long-transition-region tapered fiber: (**a**) 2D cross-sectional schematic, and (**b**) 3D rendered view.

To create multiple spatially separated potential wells in the far field, a model with a long transition region and an extended waist was designed (Figure 12). The model used a standard single-mode fiber (cladding diameter 125 μm, core diameter 10 µm) with a transition length of approximately 266 µm, a waist length of approximately 454 µm, and a waist diameter of approximately 48 µm. The structure was imported into an FDTD solver and illuminated at wavelengths of 900, 950, 960, and 980 nm.

**Figure 13:**
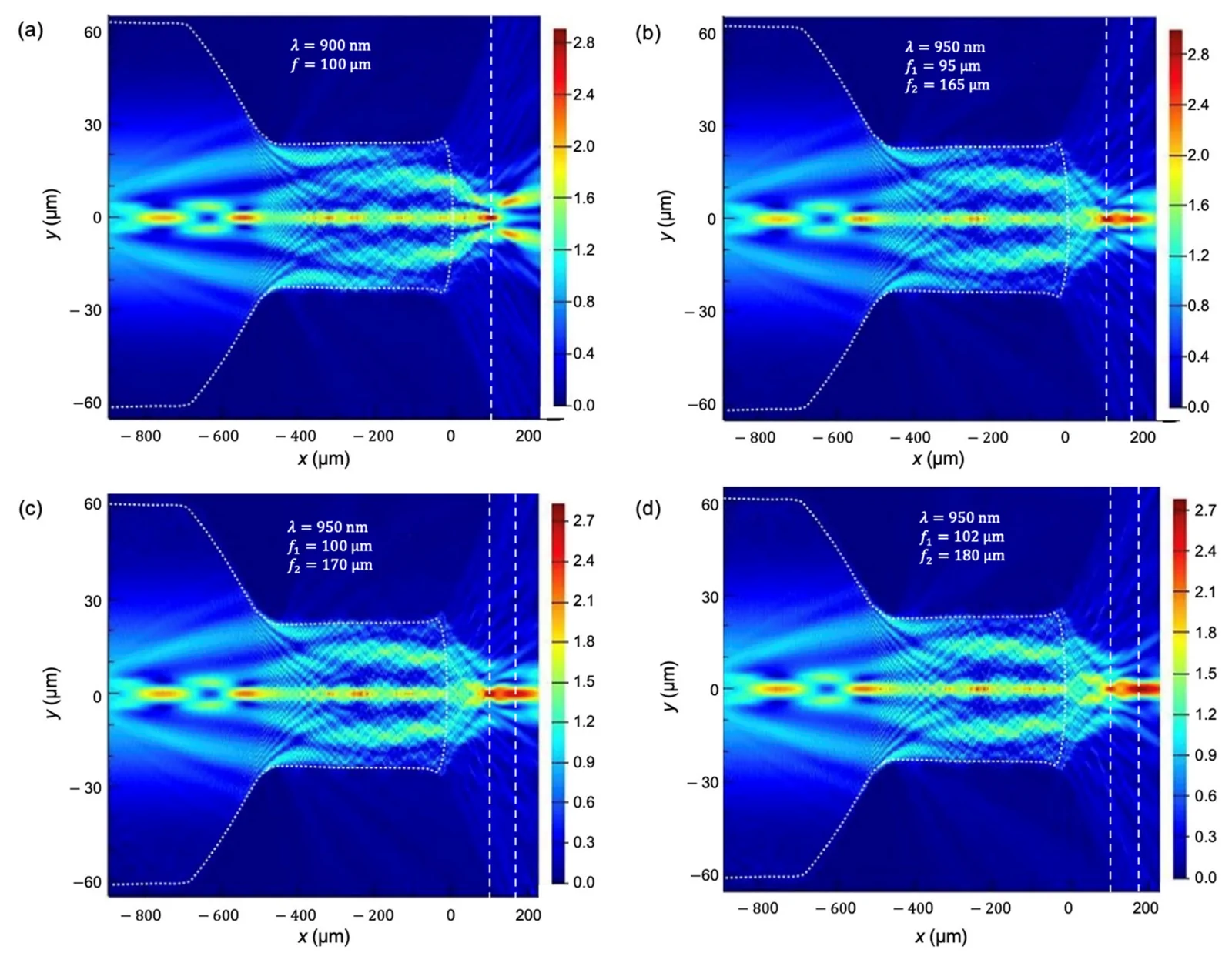
Dual-focus distributions of the long-transition-region tapered fiber at different incident wavelengths: (**a**) 900 nm; (**b**) 950 nm; (**c**) 960 nm; (**d**) 980 nm. Vertical dotted lines represent the foci.

FDTD Simulations (Figure 13) reveal a single axial intensity peak near 100 µm when the incident wavelength is 900 nm. As the wavelength increases beyond 950 nm, this peak splits into two distinct foci whose axial positions shift with wavelength. At 950 nm, the foci appear near 95 µm and 165 µm; at 960 nm, near 100 µm and 170 µm; and at 980 nm, near 102 µm and 180 µm. Notably, the second focus shifts more rapidly with wavelength, thereby enlarging the separation between the two high-intensity regions. This wavelength-dependent divergence provides the optical-field basis for dual-well trapping.

**Figure 14:**
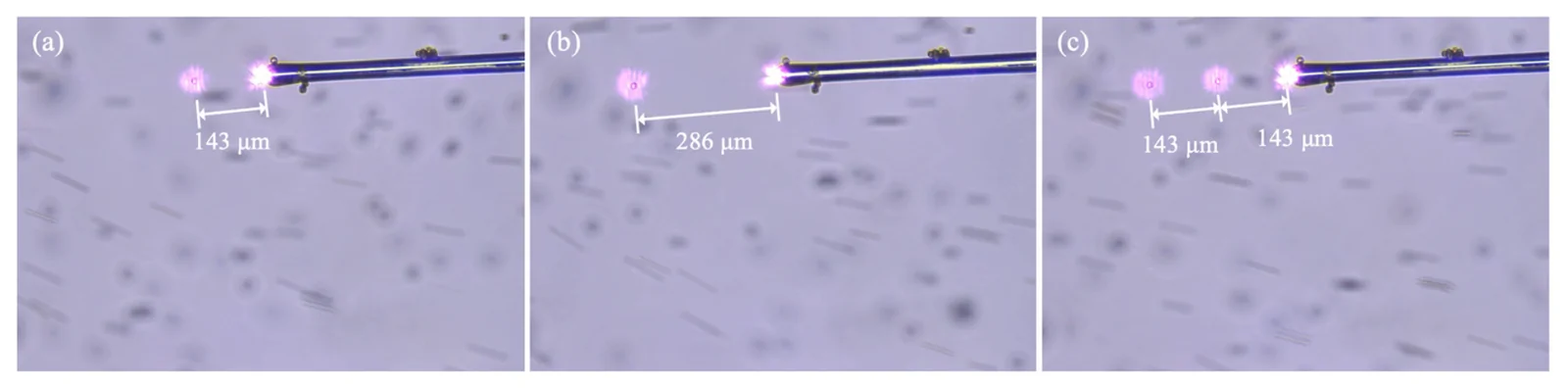
Dual-well trapping of 5 µm PS microspheres at 980 nm and powers above 500 mW: (**a**) trapping at approximately 143 µm; (**b**) simultaneous occupation of both potential wells; (**c**) trapping at approximately 286 µm.

Experimental validation used a 980 nm laser and 5 µm PS microspheres. At input powers above 500 mW, microspheres were stably trapped at approximately 143 µm or 286 µm from the end face. Both axial positions could be occupied simultaneously (Figure 14). Particle positions remained essentially unchanged whether a single potential well or both potential wells were occupied, confirming the formation of spatially separated, effective far-field potential wells along the optical axis. Because the two potential wells are separated by more than 140 µm, the presence of one trapped microsphere does not appreciably perturb the optical field at the other site. Both trapping positions are defined by the dual-focus distribution of the tapered fiber itself, in contrast to the flame-tapered case, where microlens effects from the first bead generate a secondary near-tip potential well.

These results show that machine-pulled tapered fibers with lens-shaped end faces and long transition regions provide a reproducible route to extend optical interaction distances from micrometers to hundreds of micrometers. Parabolic end-profile design enables tuning of focal distance and axial confinement, and appropriately engineered structures support single- and dual-well far-field trapping. These capabilities complement the strong near-tip confinement of flame-tapered probes and expand the operational range for fiber-based optical manipulation.

## 4. Modulation of the Trapping Distance in Different Media

### 4.1. Effect of the Medium Refractive Index on the Focal Position

The preceding sections show that fiber geometry can extend the fundamental working range from several micrometers near the end face to hundreds of micrometers. Once a probe has been fabricated, however, its transition region and end-face curvature are fixed. Adjusting the optical power generally changes only the potential-well depth and trapping stability and cannot independently and continuously move the focus. To tune the trapping position with a fixed structure, the fiber model and incident conditions were held constant while only the refractive index of the external medium was changed. The output fields in water (*n* = 1.33) and ethanol (*n* = 1.36) were then compared.

**Figure 15:**
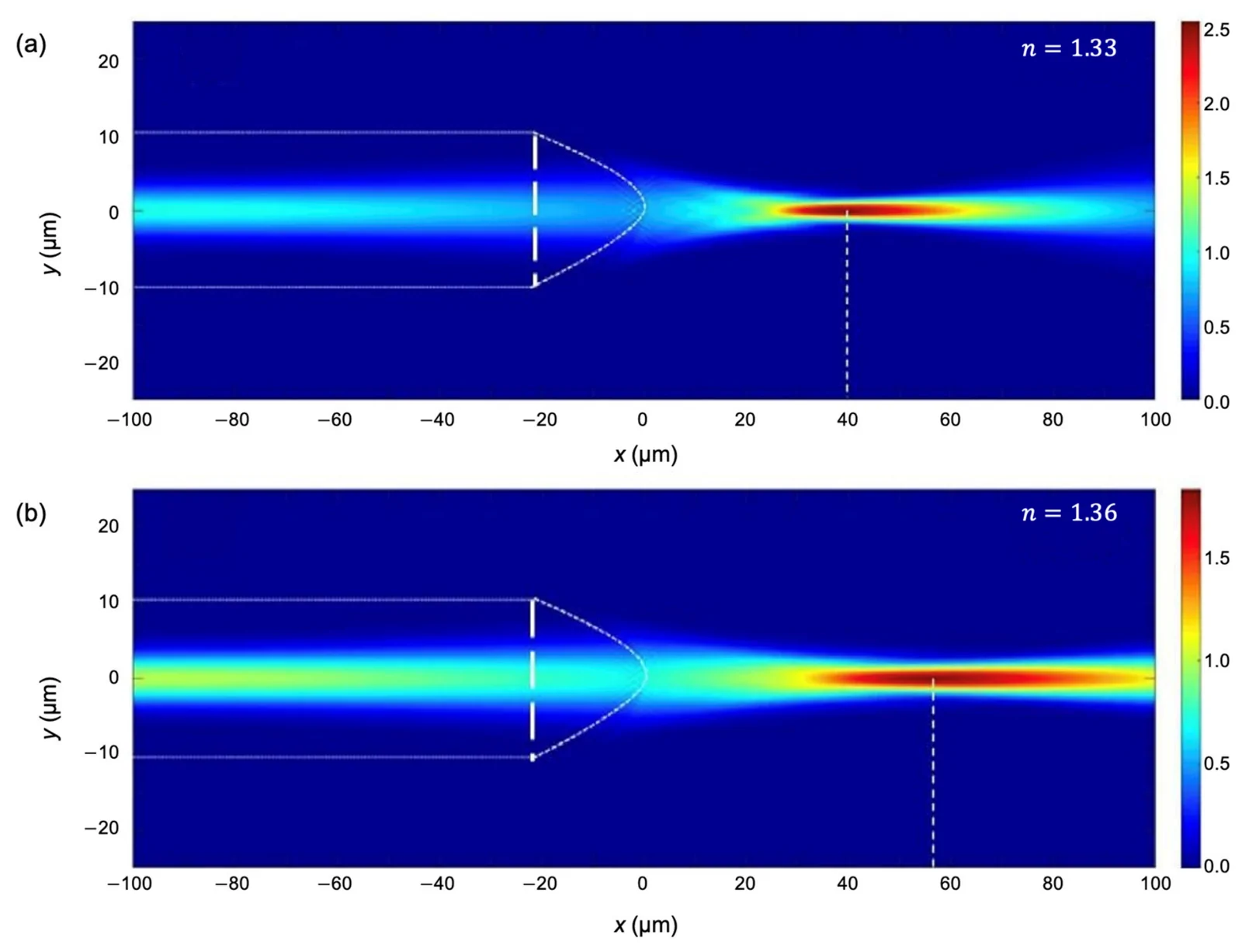
Output fields at different external refractive indices: (**a**) water, *n* = 1.33, with a focal position of approximately 40 µm; (**b**) ethanol, *n* = 1.36, with a focal position of approximately 55 µm.

As shown in Figure 15, the simulations show that increasing the external refractive index shifts the focal position away from the end face. Specifically, the primary focus moved from approximately 40 µm in water to 55 µm in ethanol, a shift of about 15 µm. This behavior arises from the change in relative index contrast at the fiber–medium interface: as the external refractive index increases, the index contrast decreases, reducing refraction at the interface and producing a less convergent wavefront at the fiber exit. The result is a focal region that forms farther from the tip and, depending on the contrast, may also change in peak intensity and spatial extent. Thus, the medium refractive index directly controls not only the magnitude of the optical forces on a particle but also the axial location of the potential well produced by a fixed fiber structure.

### 4.2. Variation in Trapping Distance During Ethanol Evaporation

**Figure 16:**
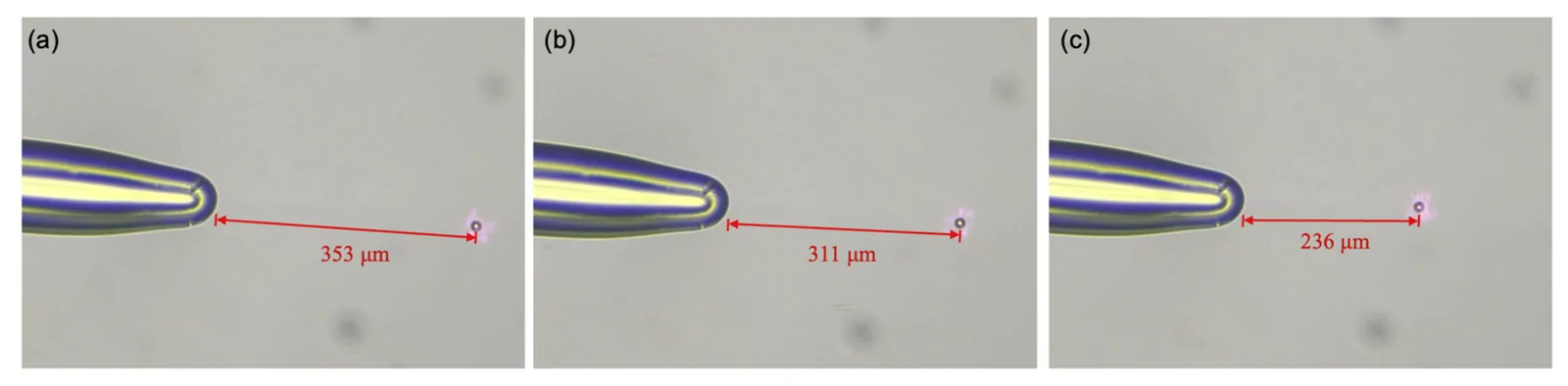
Movement of the trapping position toward the fiber end face during ethanol–water evaporation: (**a**) 353 µm; (**b**) 311 µm; (**c**) 236 µm. The panels are arranged chronologically.

To experimentally validate the modulation of the trapping position by the surrounding environment, an evaporation experiment was conducted on an ethanol–water droplet, and the distance between the stably trapped particle and the fiber end face was recorded over time. During evaporation of an ethanol–water binary droplet, the preferential loss of the more volatile component changes the liquid composition with time. Previous measurements using a refractive-index–ethanol-concentration calibration have directly confirmed this compositional evolution in evaporating ethanol–water droplets [36]. At the beginning of the experiment, the ethanol fraction was relatively high, and the solution’s refractive index was closer to that of ethanol; the particle was then stabilized approximately 353 µm from the end face. As ethanol evaporated, the trapping position moved to approximately 311 µm. When the liquid composition approached that of water, the stable position shifted further to approximately 236 µm (Figure 16).

This unidirectional inward shift is consistent with the simulation results: decreasing the external refractive index increases the fiber–medium index contrast, strengthens refraction at the exit, and pulls the focal region and associated potential well closer to the tip. The observed continuous change from 353 to 236 µm demonstrates that environmental composition can serve as an effective passive-to-active control parameter for tuning the trapping distance over a range of tens to hundreds of micrometers.

Combining structural design and environmental tuning (Section 2, Section 3 and Section 4) yields a practical multiscale manipulation strategy: fiber geometry selects the basic trapping range, and the surrounding refractive index tunes the equilibrium position within that range. Flame-tapered tips provide strong near-tip confinement within several micrometers, while machine-pulled long-transition probes extend the interaction range to hundreds of micrometers. Within either range, modest changes in the external refractive index shift the focal position and the stable trapping site, enabling continuous, structure-independent adjustment of trapping distance without mechanical modification of the probe.

## 5. Conclusions

This work demonstrates a practical, multiscale approach to fiber-based optical manipulation that combines tailored fiber geometry with environmental refractive-index control to achieve continuous tuning of the trapping distance from the near-field to the hundred-micrometer scale. Flame-tapered tips produce strong, micrometer-scale convergent fields immediately in front of the end face, enabling reliable single-particle trapping and probe-guided transport; simulations and experiments for taper angles of 24°, 40°, and 60° locate primary foci at approximately 0.2 µm, 5.1 µm, and 7.0 µm, respectively, and identify a stable operating power window near 84–120 mW beyond which thermal and hydrodynamic disturbances degrade confinement. A trapped microsphere reshapes the downstream field through refraction, scattering, and a microlens effect, creating a secondary high-gradient region capable of capturing a second particle; this secondary potential well is inherently more sensitive to power, particle decentering, interparticle spacing, and flow than the primary near-tip trap.

Machine-pulled tapered fibers with long, smoothly curved transition regions and lens-shaped end faces extend the interaction range to the hundred-micrometer regime. Parabolic end-profile simulations show that increasing end curvature lengthens the focal distance, broadens the focal region, and reduces peak intensity. Experiments confirm reproducible trapping at tens to hundreds of micrometers (representative distances of approximately 67 µm, 122 µm, and 163 µm). A long-transition design further supports dual-focus far-field trapping, with two spatially separated potential wells whose separation increases with wavelength; at 980 nm and elevated input power, two stable potential wells were observed experimentally at roughly 143 µm and 286 µm from the end face.

Crucially, with the probe geometry fixed, the surrounding medium provides an effective, continuous control knob for the equilibrium trapping position. Simulations indicate that modest increases in external refractive index shift the focal position away from the tip (for example, from approximately 40 μm at *n* = 1.33 to 55 µm at *n* = 1.36), and evaporation experiments in ethanol–water droplets show a progressive inward migration of a trapped particle from ~353 µm to ~311 µm and then to ~236 µm as composition changes. These results validate the central design principle of this study: fiber geometry establishes the accessible trapping range, while the medium refractive index tunes the precise trapping location within that range.

The combined strategy yields a compact, insertable platform for precise near-tip manipulation, remote positioning at the hundred-micrometer scale, and composition-sensitive control of trapping sites, with immediate relevance to particle handling and biological manipulation in confined or microfluidic environments. Future work should quantify photothermal and hydrodynamic coupling across media, perform experimental trap-stiffness calibrations to quantitatively validate theoretical force simulations, optimize end-face geometries to maximize far-field intensity while minimizing heating, and integrate refractive-index control with microfluidic delivery to enable automated, reversible distance tuning for advanced lab-on-a-chip applications.

## Figures and Tables

**Table 1:** Geometrical parameters of the three alcohol-lamp-tapered probes.

Probe	Taper Angle (°)	Tip Diameter (µm)	Taper Length (µm)
A	24	7	280
B	40	9	125
C	60	15	95

**Table 2:** Trapping performance of 20 µm PS microspheres at different laser powers.

Laser Power (mW)	Trapping State	Particle Behavior
60	No trapping	The optical force is insufficient to draw the microsphere into the trap.
78	Weak trapping	The microsphere exhibits large fluctuations and readily escapes.
84	Stable trapping	The stable-trapping threshold is reached.
100	Stable trapping	The particle remains stably confined.
120	Stable trapping	The particle remains stably confined.
122	Critical	Thermal effects begin to emerge and the motion amplitude increases.
130	Unstable	The microsphere oscillates strongly and escapes after approximately 10 s.
140	Escape	The microsphere rapidly leaves the optical trap.

## Data Availability

Raw data presented in this study are available on request from the corresponding author.

## Supplementary Materials

The following supporting information can be downloaded at https://www.mdpi.com/article/10.3390/bios16100557/s1, Movie S1: PS Bead Trapping, Movie S2: High Power Regime, Movie S3: Dual Particle Trapping.
